# Regulation of COP1 Function by Brassinosteroid Signaling

**DOI:** 10.3389/fpls.2020.01151

**Published:** 2020-07-31

**Authors:** Cristina Nieto, Luis Miguel Luengo, Salomé Prat

**Affiliations:** Department of Plant Molecular Genetics, Centro Nacional de Biotecnología (CNB-CSIC), Madrid, Spain

**Keywords:** thermomorphogenic elongation, COP1, brassinosteroid, cop1-6, Arabidopsis

## Abstract

Small increases in temperature result in enhanced elongation of the hypocotyl and petioles and hyponastic growth, in an adaptive response directed to the cooling of the leaves and to protect the shoot meristem from the warm soil. This response, collectively termed as thermomorphogenesis, relies on the faster reversion of phyB Pfr at warmer temperatures, which leads to enhanced activity of the basic-helix-loop-helix PHYTOCHROME INTERACTING FACTOR 4 (PIF4). PIF4 acts as a molecular hub integrating light and temperature cues with endogenous hormonal signaling, and drives thermoresponsive growth by directly activating auxin synthesis and signaling genes. Growth promotion by PIF4 depends on brassinosteroid (BR) signaling, as indicated by the impaired thermoresponse of BR-defective mutants and the partial restoration of *pifq* thermoresponsive defects by brassinolide (BL) application. Also, phyB limits thermomorphogenic elongation through negative regulation of the E3 ubiquitin ligase COP1 that triggers nuclear degradation of multiple photomorphogenesis-promoting factors acting antagonistically to PIF4. COP1 is indeed observed to accumulate in the nucleus in darkness, or in response to warm temperatures, with constitutive photomorphogenic *cop1* mutants failing to respond to temperature. Here we explored the role of BR signaling on COP1 function, by growing *cop1* seedlings on BL or the inhibitor *brassinazole* (BRZ), under different light and temperature regimes. We show that weak *cop1* alleles exhibit a hyposensitive response to BL. Furthermore, while *cop1-6* mutants display as described a wild-type response to temperature in continuous darkness, this response is abolished by BRZ. Application of this inhibitor likewise suppressed temperature-induced COP1 nuclear accumulation in *N. benthamiana* leaves. Overall these results demonstrate that *cop1-6* is not a temperature-conditional allele, but this mutation allows for a partially active protein which unveils a pivotal role of active BR signaling in the control of COP1 activity.

## Introduction

Light and temperature are key environmental factors regulating plant growth and development. In nature, the hypocotyl of dark germinated seedlings rapidly elongates and folds in an apical hook, while the cotyledons remain closed and unexpanded to facilitate shoot emergence from soil, in the so-called skotomorphogenesis process. On light perception, photomorphogenesis is initiated and hypocotyl growth is suppressed, at the time that cotyledons expand and chlorophyll is produced. Warm temperatures produce morphological changes in the light, which contribute to protect the shoot meristem from the warm soil and to avoid heat-induced damage of the photosynthetic machinery (Quint et al., 2016). This adaptive response, known as thermomorphogenesis, leads to elongation of the hypocotyl and the petioles, and hyponastic growth, and relies on the ability of plants of integrating external light and temperature cues with the inner circadian clock, to the coordinated regulation of endogenous plant hormones such as auxin, gibberellin and brassinosteroids (Casal and Balasubramanian, 2019; Gil and Park, 2019).

Phytochrome-mediated light responses have been extensively linked to a direct regulatory effect on the family of basic-helix-loop-helix transcription factors *PHYTOCHROME INTERACTING FACTORS* (PIFs) (Pfeiffer et al., 2014). Light-induced nuclear translocation of phytochrome B (phyB) mediates phosphorylation and subsequent degradation of these regulators that function as central hubs of a hypocotyl elongation regulatory cascade leading to direct activation of the auxin biosynthetic *TAA1* and *YUCCA* genes, in addition to the modulation of multiple cell wall loosening enzymes with a role in directional cell expansion (Zhang et al., 2013).

Different molecular factors with a role in thermomorphogenic elongation have been identified to date in *Arabidopsis.* Among those, phyB is established to act as a main thermosensor, as increases in ambient temperature lead to faster Pfr reversion and reduced photoreceptor activity (Jung et al., 2016; Legris et al., 2016). PIF4 was shown to accumulate at elevated temperatures (Foreman et al., 2011) and to promote themormorphogenic elongation *via* direct activation of auxin biosynthetic and signaling genes (Koini et al., 2009; Nozue et al., 2011), in addition to the up-regulation of BR levels *via* direct interaction with the BR signaling BZR1 factor (Oh et al., 2012). PIF4-BES/BZR1 complex formation switches BES1/BZR1 transcriptional activity from a repressive into an activator function and leads to de-repressed expression of BR-biosynthetic genes (Martínez et al., 2018). Impaired thermoresponse of BR biosynthetic mutants (Ibañez et al., 2018), along with restoration of the thermoresponsive defects of *pifq* mutants by BL (Martínez et al., 2018), indeed shows that functional BR signaling is essential to thermomorphogenic elongation. Perception of BRs by the BRASSINOSTEROID INSENSITIVE 1 (BRI1) receptor promotes its interaction with the co-receptor BRI1-ASSOCIATED RECEPTOR KINASE 1 (BAK1) and triggers a phosphorylation cascade that leads to inactivation of the BRASSINOSTEROID INSENSITIVE 2 (BIN2) kinase, with a master negative regulatory role in BR signaling (Belkhadir and Jaillais, 2015). Inactivation of BIN2 allows PP2A-mediated de-phosphorylation of BES1/BZR1 and nuclear accumulation of these factors (Tang et al., 2011), in addition to suppress BIN2-dependent phosphorylation and destabilization of PIF4 (Bernardo-García et al., 2014). BZR1 accumulates into the nucleus in response to warm temperatures, and binds the *PIF4* promoter to its activated expression (Ibañez et al., 2018).

phyB moreover suppresses the activity of the ring-finger E3 ubiquitin ligase CONSTITUTIVE PHOTOMORPHOGENIC 1 (COP1), and this negative control depends on light and temperature signaling (Ma et al., 2002; McNellis et al., 2007; Park et al., 2017). COP1 activity is increased in the dark and under warm temperature regimes, where the COP1 protein is observed to preferentially accumulate in the nucleus. COP1 targets for degradation multiple photomorphogenesis-promoting transcription factors acting antagonistically to PIF4, such as ELONGATED HYPOCOTYL (HY5) (Ang et al., 1998). Notably, COP1 function is described to enhance *PIF4* expression (Gangappa et al., 2017) and promote PIFs activity by binding and inhibiting the BR signaling kinase BIN2 (Bernardo-García et al., 2014; Ling et al., 2017), in addition to ubiquitinate and target for degradation the inactive phosphorylated form of BZR1 (Kim et al., 2014). Accordingly, the constitutive photomorphogenic *cop1* mutants fail to respond to warm temperatures.

Here, we show that weak *cop1* mutants display a hyposensitive response to BL treatment and that application of the inhibitor *brassinazole* (BRZ) suppresses the thermosensitive phenotype of *cop1-6* alleles. Remarkably, *cop1-6* mutants exhibit a wild-type thermoresponse in darkness, but fail to respond to temperature under diel conditions. This indicates that the in frame full length cryptic splicing form of *cop1-6* retains part of COP1 activity, this mutation thus providing an excellent tool to the study of the role of BR signaling in the temperature-dependent control of COP1.

## Materials and Methods

### Plant Material and Growth Conditions

Wild type *Arabidopsis thaliana* Col-0 plants and *cop1-4* and *cop1-6* mutants in the Col-0 background (McNellis et al., 1994) were used in this study. Seeds were surface-sterilized for 15 min in 70% (v/v) ethanol and 0.01% (v/v) Triton X-100, followed by two washes of 2 min in 96% (v/v) ethanol. Seeds were air dried and sown on half strength MS-agar plates with 1% sucrose and stratified for 3 days at 4°C in the dark. Germination was synchronized by illuminating the plates for 4 h and transferring them back to darkness for 20 additional hours. Seedlings were grown in darkness or in short days (8 h light/16 h dark) under 50 µmol∙m^−2^∙s^−1^ fluorescent white lights, in light chambers maintained at 22°C or 28°C.

### Hypocotyl Measurements

To measure hypocotyl growth, seeds were sown on half strength MS-agar plates with 1% sucrose, supplemented with the BL/BZR treatments in vertical plates. After 5 days, seedlings (n = 15–30) were photographed and the length of the hypocotyls was measured with the ImageJ software.

### Transient Expression in *N. benthamiana*

*Agrobacterium tumefaciens* AGL21 containing the respective constructs and the p19-silencing suppressor were grown overnight at 28°C in YEB medium supplemented with the appropriate antibiotics. Cultures were pelleted and suspended in 10 mM MES pH 5.5, 10 mM MgSO4 and 150 μM acetosyringone at a OD_600_ = 0.5. The constructs and p19 cultures were mixed in a 1:1 ratio and used for infiltration of 4 to 5 leaf-old *N. benthamiana* plants. Protein accumulation was observed 2 to 3 dpi. For BRZ treatments, leaves were infiltrated with mock solution or 4 µM epiBL, 24 h before confocal microscopy observation.

### RNA Isolation and Quantitative RT-PCR Analysis

Total RNA from 5 days old seedlings was extracted using the High Pure RNA Isolation kit (Roche) according to the manufacturer’s instructions. 1 µg total RNA was used for cDNA preparation, using the Transcriptor First Strand cDNA synthesis kit (Roche). Quantitative RT-PCR analyses, using the FastStart Universal SYBR Green Master (Roche), were performed using the 7500 Real-Time PCR System (Applied Biosystems). Each sample was analyzed in quadruplicate. The gene specific primers for *COP1* and *cop1-6 T1-T4* amplification were: COP1-Fw (5′-GAGCACCGGATCTGGATAAA-3′); COP1-Rev (5′-ACATGCCGAGCTGAAGTTTT-3′), cop1-6T1-Fw (5′-AACGGATTCATGCTCAGAATGTT-3′); cop1-6T2-Fw (5′-AACGGATTCATGCTCAGTGTCTTG-3′); cop1-6T3*-*Fw (5′-AACGGATTCATGCTCA GATTGC-3′); cop1-6T-Rev (5′-CCGTTGCTATAGCCTTCCCTCC-3′) cop1-6T4-Fw (5′-GGCCCCAGTGATTGTGTTAC-3′); cop1-6T4-Rev (5′-TGAAGTCCAATCGGCTTTTT-3′). Transcript T3 was observed to be generated from an AG acceptor site adjacent to the one initially described (McNellis et al., 1994). T3 actually includes a 35-bp insertion and primer cop1-6T3-Fw was modified accordingly. The *PP2A* gene was used as endogenous control to normalize target gene expression between samples, by using primers PP2A-Fw (5′-TAACGTGGCCAAAATGATGC-3′) and PP2A-Rev (5’-GTTCTCCACAACCGCTTGGT-3′).

### Semi-Quantitative PCR

A *cop1-6* cDNA was used as template to analyze the relative abundance of T1-T4 transcripts by PCR amplification with the TaqI DNA Polymerase (Biotools), using 35 cycles of 94°C/15 s; 55°C/30 s; 72°C/20 s; and a final extension at 72°C/7 min. The PCR amplification products were separated on a 3% agarose gel. Specific primers used were cop1-6T-Fw (5′-GAAAGAAACGGATTCATGCTCAG-3′) and cop1-6T-Rev.

### Plasmid Constructs

The full-length *COP1*, *cop1-6 T2*, and *cop1-4* coding sequences (CDS) were cloned into the Gateway System (pENTR™ Directional TOPO^®^ Cloning kit and Gateway^®^ LR Clonase II Enzyme Mix, Invitrogen) following the manufacturer recommendations. *COP1* and *cop1-4* CDS were amplified using the forward primer *COP1-*cloning Fw (5′-caccATGGAAGAGATTTCGACGGATC-3′) and the reverse primers *COP1*-cloning Rev (5′-GAagatctTCACGCAGCGAGTACCAGAAC-3′) and *cop1-4*-cloning Rev (5′-TTACGAATCTGACCCACTCAGCGCATCC-3′), respectively. To amplify *cop1-6 T2*, the 15 bp insertion in this transcript was introduced by overlapping PCR, using the following pairs of primers: *COP1-*cloning Fw/*cop1-6*-cloning Rev (5′CCACAAGACAAGACACTGAGCATGAATCCGTTTCTTTCTAGCC-3′) and *cop1-6*-cloning Fw (5′-CAGTGTCTTGTCTTGTGGTTCAATGATTTACAAGAATGTTA-3′)/*COP1-*cloning Rev. The binary vector pUBN-RFP-Dest (Grefen et al., 2010) was used for N-terminal terminal fusion of the mRFP tag.

### Confocal Microscopy

Fluorescence imaging was performed on a Leica TCS-SP8 multispectral confocal system equipped with the LAS X v. 2.0 control software (Leica Microsystems), gated-hybrid emission detectors, and a white laser beam (WLL2) for 470 to 670 excitation, by using a 63x/1.20NA water immersion objective. The 555 nm and 640 nm laser lines were used for RFP (in red) and chloroplast excitation (in blue). The emission bandwidths between 565 to 630 nm and 654 to 748 nm were used for RFP and chloroplast detection respectively.

## Results

### Darkness and Warm Temperatures Suppress the Short Hypocotyl of *cop1-6* Seedlings

COP1 is a central repressor of photomorphogenesis (Deng et al., 1991) and, consistent with this function, *cop1* mutants display a constitutive photomorphogenic phenotype in the dark. Several independent *cop1* recessive mutations have been described and classified in three phenotypic classes: weak, strong, and lethal (McNellis et al., 1994). Weak *cop1-4* and *cop1-6* alleles display slightly longer hypocotyls in dark conditions as compared to the light, suggestive of a residual activity of these proteins (McNellis et al., 1994)

**Figure 1:**
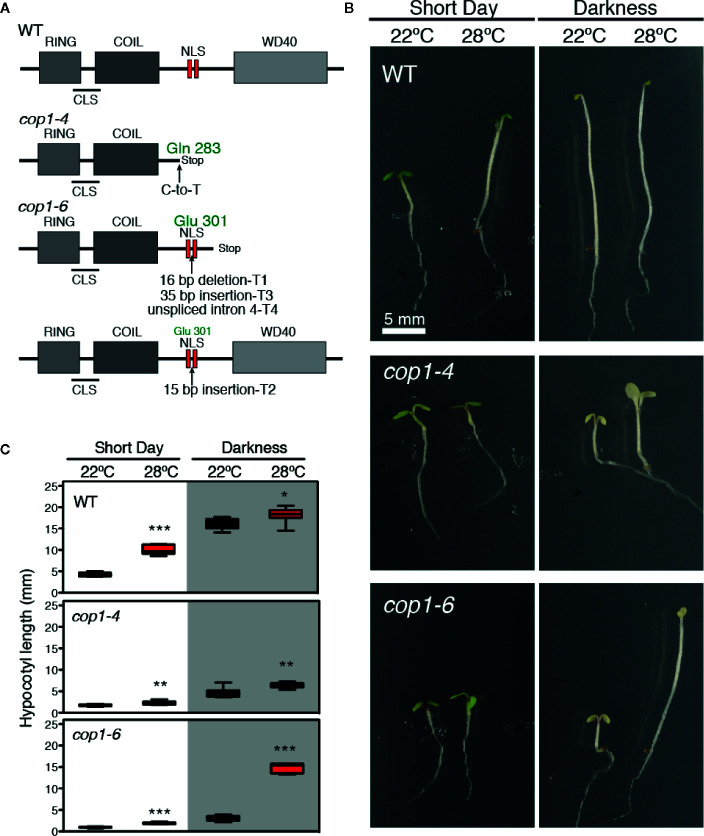
Darkness and warm temperatures rescue the photomorphogenic phenotype of *cop1-6* mutants. **(A)** COP1 structural domains. CLS and NLS are the cytoplasmic and nuclear localization signals, respectively. The *cop1-6* allele carries a point mutation at the acceptor site of intron 4 that causes three cryptically spliced transcripts (T1-T3) and a transcript with the unspliced intron 4 (T4). T2 encodes a full-length protein with 5 extra aa between the two NLS. The *cop1-4* allele is a point mutation, resulting in a truncated protein (adapted from McNellis et al., 1994). **(B)** Visible hypocotyl phenotypes of Col-0, *cop1-4* and *cop1-6* mutants. Seedlings were grown for 5 days in constant dark/SD and at 22°C or 28°C. **(C)** Box plots showing the hypocotyl length measurements. Error bars indicate standard deviation (n=15 - 30). The data were statistically analyzed using a two-tailed Student’s t-test (*p < 0.05, **p < 0.001, **p < 0.0001, 28°C compared with 22°C conditions).

In the *cop1-6* allele (McNellis et al., 1994), a point mutation at the intron 4 acceptor splicing junction causes the cryptic splicing into three mRNAs (T1-T3), along with the unspliced intron 4 transcript (T4), as shown in **Figure 1A**. T1, T3, and T4 lead to truncated proteins after Glu-301, which are the result of a 16-bp deletion, a 35-bp insertion, and the unspliced intron 4, respectively. By opposite, T2 produces a full-length in frame protein with a 5 amino acids insertion after Glu-301, between the two nuclear localization signals (NLS) (**Figure 1A**). The *cop1-4* allele corresponds to a C-to-T mutation which changes Gln-283 into a stop codon, leading to a truncated protein lacking the two NLS and the C-terminal WD-40 repeat domains. Notably, the *cop1-6* allele was previously described as a temperature-conditional mutation (Hsieh et al., 2000), given that this mutant exhibits a constitutively photomorphogenic development in the dark when grown at temperatures below 28°C, but elongated hypocotyls above 28°C.

To gain deeper insight into the effects of light and temperature on these two mutant alleles, we grew WT, *cop1-4* and *cop1-6* seedlings for 5 days at 22°C or 28°C, in either short days (SD) or constant dark conditions, and measured their hypocotyl lengths. WT plants showed the expected thermomorphogenic phenotype, with elongated hypocotyls at 28°C under short day cycles, and long hypocotyls with small pale cotyledons in darkness, in contrast to *cop1-4* and *cop1-6* seedlings which at 22°C, displayed a constitutive photomorphogenic phenotype both in SD conditions and in continuous dark. Dark grown *cop1-6* seedlings, however, developed elongated hypocotyls and smaller cotyledons at 28°C, in opposite to the de-etiolated phenotype of *cop1-4* mutants (**Figures 1B, C**).

### *cop1-6 T2* Expression Levels Are Independent of Light and Temperature Conditions

**Figure 2:**
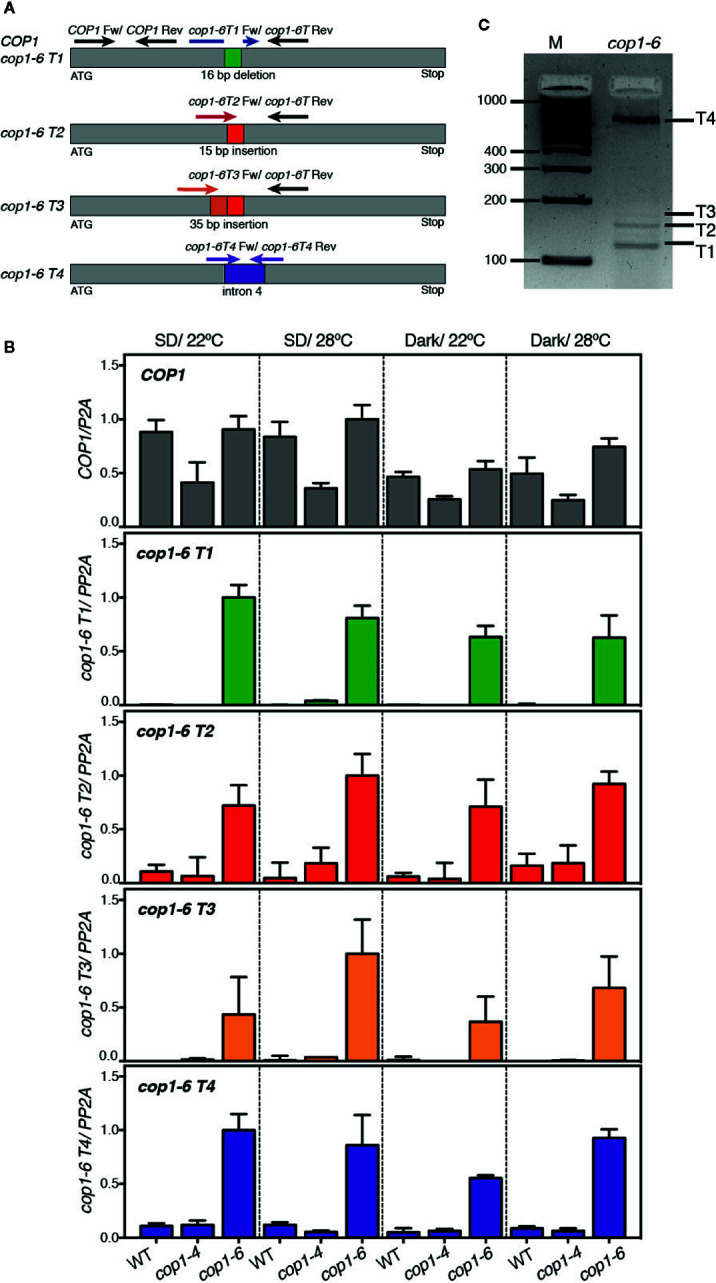
Analysis of expression levels of the *cop1-6 T1-T4* transcripts. **(A)** Diagram showing the positions of primers used for quantitative RT-PCR analysis of *cop1-6 T1-T4* and *COP1*. **(B)** *cop1-6 T1-T4* transcripts were detected in the *cop1-6* mutant but not in the Col-0 and *cop1-4* backgrounds. Total RNA was extracted from 5-day-old seedlings. *PP2A* was used to normalize gene expression levels. Relative levels are the relative quantification (RQ) mean of four technical replicates. Error bars indicate ± RQ maximum and minimum. **(C)** PCR products amplified from the *cop1-6* cDNA using primers to 5’ and 3’-regions near the point mutation. PCR products were separated on a 3% agarose gel. M indicates the size of the molecular marker in base pairs.

Since *cop1-6* was observed to encode an active protein in dark and warm conditions, we reasoned that abundance of *cop1-6* transcript 2 (*cop1-6 T2*), coding for an in frame full-length protein, might be elevated in these conditions. To examine this possibility, we analyzed by quantitative PCR the expression levels of all four potential *cop1-*6 transcripts (T1-T4) in WT, *cop1-4* and *cop1-6* seedlings, grown in darkness or in short days, and 22°C/28°C. To this end, we designed primers specific to the different *cop1-6* insertions/deletions and to intron 4 (**Figure 2A**), and analyzed abundance of these transcripts in the different genetic backgrounds.

As shown in **Figure 2B**, an amplification signal corresponding to the *cop1-6 T1-T4* transcripts was detected in *cop1-6* seedlings, but not in the WT or *cop1-4* plants. Levels of these transcripts, however, did not significantly change in response to warm temperatures or dark conditions, showing that *cop1-6* *T2* is not elevated in the dark and 28°C. Quantification of the relative abundance of these transcripts by PCR amplification of a *cop1-6* cDNA, using primers designed on a region upstream the point mutation and on exon 5, showed that transcripts T1 and T4 are the most abundant (**Figure 2C**), while T2 and T3 accumulate to significantly lower levels, as previously described (McNellis et al., 1994).

We also examined total *COP1* expression levels in these three backgrounds to assess whether the different phenotype of *cop1-4* and *cop1-6* alleles might be caused by differences in the stability of the respective transcripts. Notably, although *COP1* mRNA levels were similar in *cop1-6* and the WT, they were reduced in the *cop1-4* background (**Figure 2B**); suggesting a reduced stability of the transcripts allowing for the truncated cop1-4 protein, or that COP1 activity is required to the indirect control of its own expression.

### *cop1-6* Mutants Display Enhanced Sensitivity to BRZ Treatments

**Figure 3:**
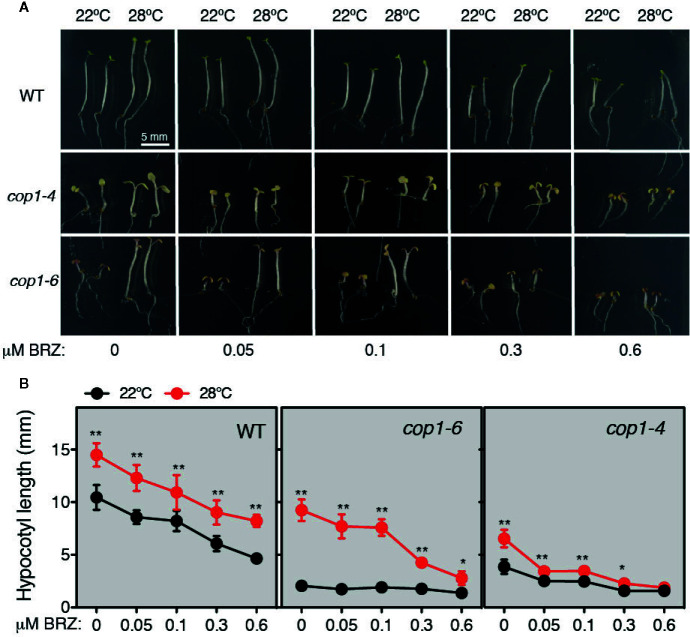
Thermo-induced hypocotyl elongation of *cop1-6* seedlings is drastically reduced upon BRZ treatment. **(A)** Representative pictures of 5 days-old Col-0, *cop1-6* and *cop1-4* seedlings grown in the dark at 22°C/28°C, in the presence of increasing BRZ concentrations. **(B)** BRZ dose-response. Graphs showing the hypocotyl lengths of 5 days-old plants grown in constant darkness and 22°C or 28°C. Error bars indicate standard deviation. (n=15 -30). Statistical analysis was performed with a two- tailed Student’s *t*-test (*p < 0.05, **p < 0.001, 28°C compared with 22°C conditions).

Thermomorphogenic elongation is recently shown to be mediated by enhanced BR synthesis (Ibañez et al., 2018; Martínez et al., 2018). The transcription factor BZR1, was shown to accumulate in the nucleus at warm temperatures and promote *PIF4* expression (Ibañez et al., 2018), leading to transcriptional activation of growth-related genes. Moreover, additional studies showed that COP1 promotes skotomorphogenesis by repressing the BR signaling kinase BIN2, that functions at PIF3 phosphorylation and degradation in the dark (Ling et al., 2017). Therefore, the observation that impaired thermomorphogenic growth of *cop1-6* mutants is rescued in continuous dark, suggested that BR signaling might regulate COP1 activity. To test this hypothesis, we studied the response of *cop1* mutants to BL or BRZ treatments, under different light and temperature regimes. First, we grew WT, *cop1-4* and *cop1-6* seedlings on MS medium containing different concentrations of BRZ (0, 0.05, 0.1, 0.3, and 0.6 µM), in continuous dark, and either 22°C or 28°C (**Figure 3A**). Notably, *cop1-6* mutants displayed in these conditions a hypersensitive response to the inhibitor BRZ, as evidenced by a drastic reduction in temperature induced hypocotyl elongation in the dark, as compared to the WT (**Figure 3B**).

### COP1 Nuclear-Cytoplasmic Partitioning Relies on BR Signaling

**Figure 4:**
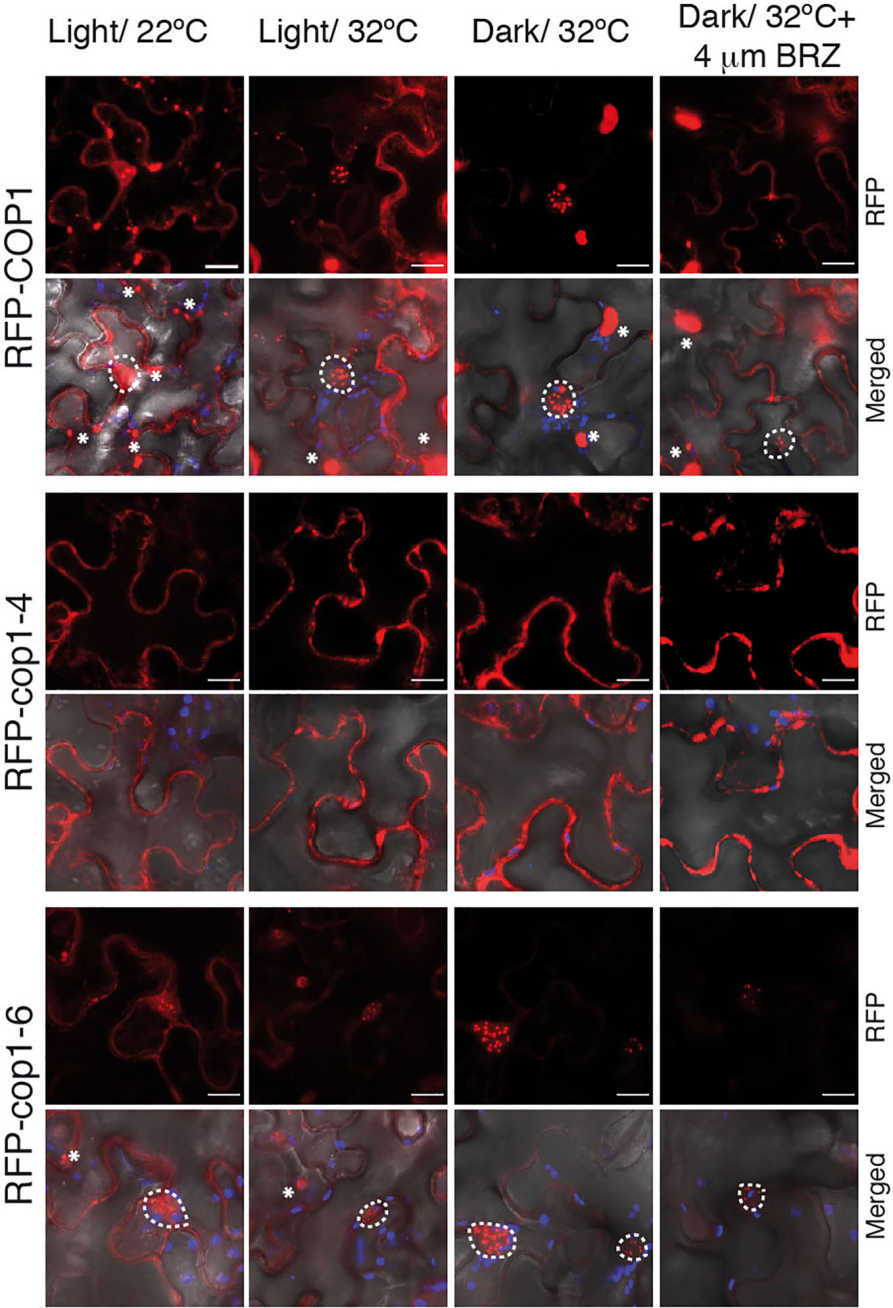
BRZ application results in nuclear depletion of COP1. Subcellular distribution of COP1 in *N. benthamiana* leaves. The RFP-tagged COP1, cop1-4 and cop1-6 proteins were agroinfiltrated in *N. benthamiana* leaves and visualized by confocal microscopy. COP1 is mainly localized in the cytoplasm and shows a diffuse nuclear signal at 22°C, in the light. After 24 h exposition to dark and warm temperatures (set to 32°C for *N. benthamiana*), COP1, and cop1-6 are shuttled to the nucleus. Leaves incubated at 32°C in the light showed a partial COP1 and cop1-6 nuclear import. Nuclear COP1 forms nuclear bodies, whereas cop1-6 has a more diffused nuclear pattern, with occasional distribution in speckles. BRZ treatment however leads to a reduction in nuclear abundance of both proteins. RFP-*cop1-4* lacks the NLS signals and is localized in the cytoplasm. RFP signal is shown in red, chloroplasts auto-fluorescence in blue and bright field in grey. Scale bars = 15 µm. Nuclear signal is delimited by white circles, whereas cytosolic bodies are indicated with asterisks.

Warm temperatures and darkness drive the nuclear localization of COP1 (Von Arnim and Deng, 1993; Park et al., 2017). To assess whether the full-length *cop1-6* protein follows the same nuclear-cytoplasmic partitioning as the WT, we analyzed the localization of N-terminal RFP fusions of the COP1, cop1-4 and cop1-6 proteins in *N. benthamiana* leaves (**Figure 4**). We first examined the subcellular localization of these proteins in leaves of plants grown in the light and 22°C. In these conditions, RFP-COP1 and RFP-cop1-6 were observed to largely accumulate in a diffuse pattern of fluorescence in the cytoplasm, in addition to aggregate into cytosolic inclusion bodies. Moreover, both of these proteins showed a residual signal in the nucleus, but whereas RFP-COP1 formed large nuclear bodies, RFP-cop1-6 displayed mainly a uniform nuclear signal with smaller speckles. RFP-cop1-4 was by opposite entirely localized in the cytoplasm (**Figure 4**).

Incubation of leaves at 28°C had no remarkable effect regarding COP1 import to the nucleus, suggesting that tobacco plants may require higher temperatures than 28°C for thermomorphogenic activation. In consequence, we incubated the infiltrated leaves for 24 h at 32°C in the dark. Remarkably, RFP-COP1 was in these conditions entirely imported into the nucleus, as it was most of the RFP-cop1-6 protein. Moreover, RFP-cop1-6 formed at 32°C similar large nuclear bodies as RFP-COP1, suggesting that this full-length variant is still able to associate into active ubiquitination complexes under dark and warm conditions, in opposite to RFP-cop1-4, lacking the NLS signals, which remained at 32°C in the cytoplasm. Light on the other hand partially suppressed nuclear import of RFP-COP1 and RFP-cop1-6. Both of these proteins showed at 32°C an intermediate distribution pattern in the light, whereby their nuclear levels were increased as compared to 22°C, but a significant signal was still detected in the cytosol (**Figure 4**). More remarkably, nuclear fluorescence by the RFP-COP1 and RFP-cop1-6 proteins was strongly reduced on application of the inhibitor BRZ (**Figure 4**). For each of these constructs signal was now stronger in the cytoplasm in correlation with a higher number of cytosolic aggregates, therefore demonstrating an effect of impaired BR synthesis in abrogating temperature-induced COP1 nuclear accumulation.

### Hyposensitive Response to BL of *cop1-6* and *cop1-4* Mutants

**Figure 5:**
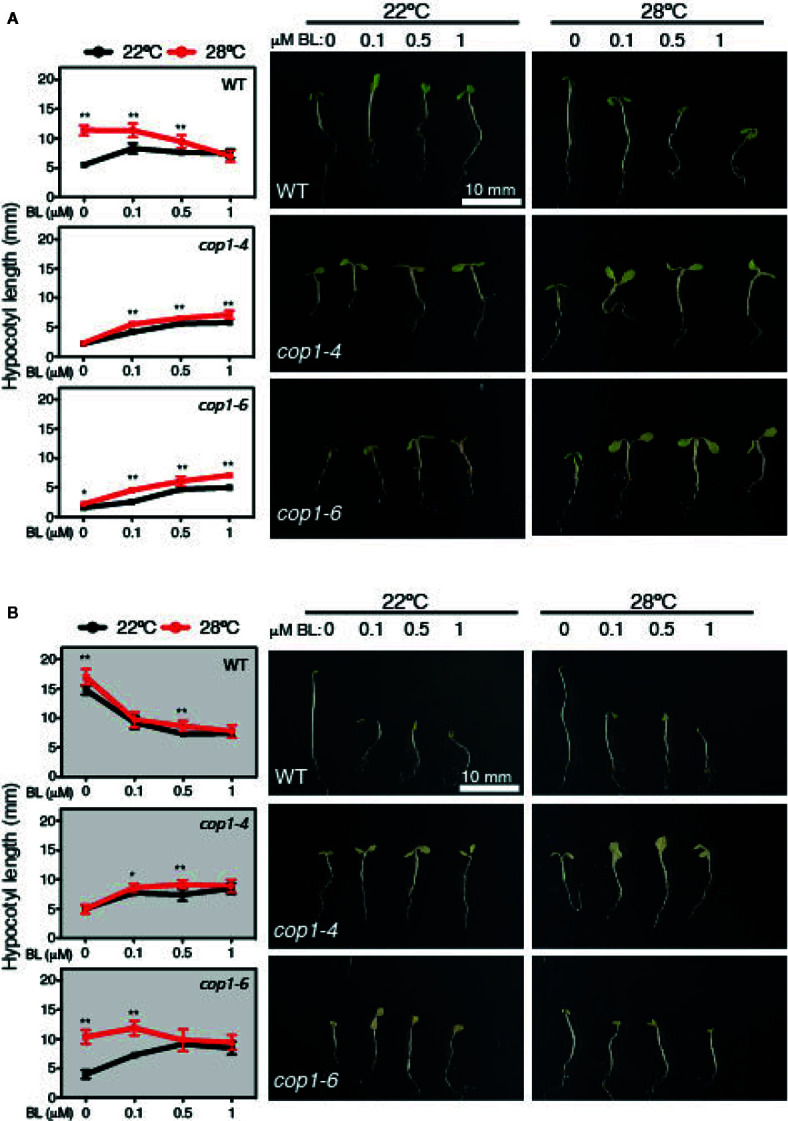
Hyposensitive response to BL treatments of *cop1* mutants. Graphs and pictures displaying the phenotypes of Col-0, *cop1-6*, and *cop1-4* seedlings grown for 5 days on different BL concentrations, under short days **(A)** and continuous dark **(B)**. Error bars indicate standard deviation (n=15). Statistical analysis was performed with a two- tailed Student’s t-test (*p < 0.05, **p < 0.001, 28°C compared with 22°C conditions).

Exogenous application of BL induces hypocotyl elongation in the light, while inhibits it under constant darkness (Oh et al., 2012). Hence, we next tested the effects of increasing BL concentrations (0, 0.1, 0.5 and 1 µM) on WT and *cop1* mutants grown under SD conditions or constant dark, at 22°C or 28°C (**Figure 5**). Notably, BL application was observed to suppress, as previously reported, hypocotyl growth of dark-grown WT seedlings. However, effects of this hormone differed in SD among seedlings grown at 22°C or 28°C. While lower concentrations of BL (up to 0.1 µm) promoted WT hypocotyl elongation at 22°C, they had a growth inhibitory effect at concentrations > 0.5 µM. Also, all concentrations of BL suppressed growth at 28°C, evidencing that warm temperatures lead to an enhanced sensitivity to BL.

Furthermore, *cop1-4* and *cop1-6* mutants displayed a reduced response to BL in SD, but a contrasting effect in continuous dark. In fact, BL application partially rescued the short hypocotyl and impaired thermoresponse of these mutants in SD, but surprisingly interfered with the temperature conditional phenotype of *cop1-6* seedlings in continuous dark (**Figure 5**). BL effects on dark grown *cop1-6* seedlings were actually comparable to those observed in SD in the WT, suggesting that impaired BR signaling of *cop1-6* is restored at elevated temperatures in the dark, in opposite to the *cop1-4* mutants that show an analogous hyposensitive phenotype in SD as in continuous dark (**Figure 5**). Therefore, these findings establish a functional link between temperature-conditional effects of the *cop1-6* mutation, BR signaling and the presence/absence of light.

## Conclusions

COP1 has been widely studied as a central repressor of light signaling and, more recently, as a thermomorphogenesis-promoting factor. Additional evidence indicates that COP1 plays a pivotal role in modulating diverse hormonal signaling pathways (Wang et al., 2019), and therefore acts as a main hub in crosstalk interaction between environmental and endogenous signals. Moreover, the COP1/SPA complex was recently established to repress BIN2-mediated phosphorylation of the PIF transcription factors (Bernardo-García et al., 2014; Ling et al., 2017), indicating that antagonism between light and BR signaling in photomorphogenic development may potentially depend on COP1 regulation of BR signaling.

Here, we show that warm temperatures suppress the photomorphogenic phenotype of *cop1-6* mutants in the dark, which suggests that the 5 amino acid insertion full-length protein translated from *cop1-6 T2* is active in these conditions, even though low expression levels of this transcript do not change at 28°C. Moreover, we observed that *cop1-6* protein activity is dependent on active BR signaling, as application of BRZ suppressed *cop1-6* hypocotyl elongation at 28°C in the dark. In these growth conditions, *cop1-6* mutants actually exhibit a hypersensitive response to BRZ, suggestive of a direct effect of BR signaling on COP1 activity. BRZ application was actually found to reduce COP1 nuclear abundance in transient expression studies in *N. benthamiana* leaves, COP1 nuclear depletion presumably leading to growth inhibition through the accumulation of HY5 and other PIFs-antagonizing factors, in addition to the reduction in PIFs protein abundance (Pham et al., 2018). Thus, it is possible that these inhibitory effects are stronger in the *cop1-6* background, because of the low abundance of T2 transcript encoding the full-length protein, although additional studies will be needed to establish whether the 5 amino acid insertion in the bipartite NLS slows down cop1-6 nuclear import rendering this protein more sensitive to BRZ inhibition.

Likewise, *cop1-4* and *cop1-6* mutants were observed to exhibit a hyposensitive response to BL, though in *cop1-6* seedlings response to this hormone was partially restored at elevated temperatures and in the dark. In fact, higher concentrations of BL disturbed hypocotyl elongation in the WT, but enhanced growth and promoted the thermoresponse of *cop1-4* and *cop1-6* mutants in SD (**Figure 5**). However, behavior of these weak alleles was radically different in continuous dark, where BL application caused at elevated temperatures a similar disturbed growth of *cop1-6* seedlings as in the WT, while temperature was of no effect on the hyposensitive response of *cop1-4* mutants (**Figure 5**). These observations demonstrate that the temperature-conditional phenotype of *cop1-6* seedlings depends on BR signaling. As such, future studies directed to analyze HY5 abundance in response to BL and BRZ application shall contribute to understand function of this pathway in modulating COP1 activity.

**Figure 6:**
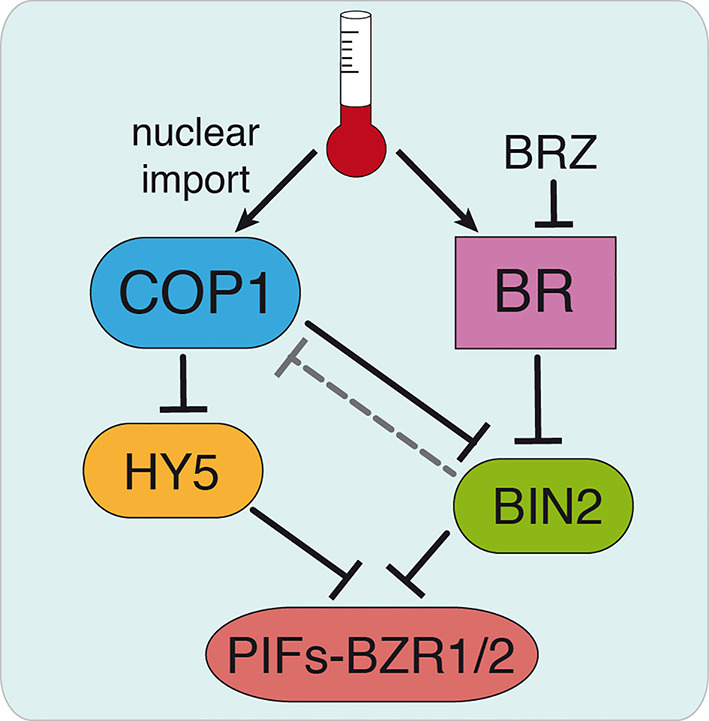
Hypothetical model for COP1 regulation by BR signaling. Thermomorphogenic hypocotyl growth is mediated by the PIF4 and BZR1/2 transcription factors and depends on active BR signaling. Warm temperatures promote COP1 nuclear shuttling and enhance PIF4 and BZR1/2 function by sequestering the BR signaling BIN2 kinase that mediates destabilization of these factors. We hypothesize that BR might exert a direct control on COP1 activity *via* BIN2, as BRZ treatments decrease COP1 nuclear abundance (dashed line). This allows HY5 accumulation, in addition to the accumulation of other factors antagonizing PIFs, and HY5-mediated restriction of hypocotyl elongation. Our findings show that cop1-6 is functional in darkness and warm temperatures, but its activity is dependent on active BR signaling. Further studies will address the mechanism by which BIN2 regulates COP1 activity, thus linking BR signaling and COP1 function.

COP1 is actually shown to promote plant growth by sequestering the BR signaling kinase BIN2, involved in PIFs and BZR1/2 phosphorylation and destabilization (Ling et al., 2017). Although the BR-biosynthesis *det2* mutant was found in initial studies to display at 22°C a similar pattern of COP1 accumulation as the WT, and BL treatments failed to increase COP1 nuclear abundance in the light (Von Arnim et al., 1997), here we show that application of the inhibitor BRZ suppresses temperature-induced COP1 nuclear accumulation. Likewise, the weak *cop1-6* allele was observed to exhibit a differential response to BL in continuous darkness or diurnal conditions at 28°C, which suggest that temperature-induced COP1 nuclear translocation enhances the response to BL, while increased BL levels are required for COP1 nuclear accumulation. These findings suggest that COP1 nuclear shuttling requires of the assistance of so far unknown light/temperature responsive components, while BR signaling is essential for nuclear COP1 stability. We hypothesize that COP1 activity may be reduced *via* BIN2-mediated phosphorylation (**Figure 6**), which would partially explain the altered response of *cop1-6* mutants to BL/BZR treatments. Future studies addressed to define the mechanisms by which BIN2 possibly regulates COP1 activity will be critical to link BR signaling and COP1 function.

## Data Availability Statement

All datasets generated for this study are included in the article/supplementary material.

## Author Contributions

CN and SP designed the study and wrote the manuscript. CN and LL performed the experiments. All authors contributed to the article and approved the submitted version.

## Funding

This work was financially supported by the Spanish Ministerio de Investigación, Economía y Competitividad (grant BIO2017-90056-R).

## Conflict of Interest

The authors declare that the research was conducted in the absence of any commercial or financial relationships that could be construed as a potential conflict of interest.
